# The mitochondrial genome of the nematode endoparasitic fungus *Hirsutella rhossiliensis*

**DOI:** 10.1080/23802359.2016.1143336

**Published:** 2016-02-10

**Authors:** Niuniu Wang, Yongjie Zhang, Muzammil Hussain, Kuan Li, Meichun Xiang, Xingzhong Liu

**Affiliations:** a State Key Laboratory of Mycology, Institute of Microbiology, Chinese Academy of Sciences, Beijing, China;; b University of Chinese Academy of Sciences, Beijing, China;; c School of Life Sciences, Shanxi University, Taiyuan, China

**Keywords:** *Hirsutella rhossiliensis*, Hypocreales, mitochondrial genome, Ophiocordycipitaceae

## Abstract

In this study, we report the complete mitochondrial genome of *Hirsutella rhossiliensis* (Ophiocordycipitaceae, Hypocreales, Ascomycota). We construct the mitochondrial DNA genome organization of 62 483 bp in length of *H. rhossiliensis* by using the whole-genome resequencing method. Conserved genes including the large and small rRNA subunits, 26 tRNA and 14 protein-coding genes are identified. These protein-coding genes utilize ATG, GTG or TTG as initiation codons and TAA or TAG as termination codons. Moreover, we detect 10 group I introns and one unclassified intron in six genes (*rnl*, *cob*, *cox1*, *cox3*, *nad1* and *nad5*) encoding ORFs of ribosomal protein S3 and GIY-YIG/LAGLIDADG endonucleases or hypothetical proteins. This mitochondrial genome will be useful in understanding the distribution and genetic diversity of this species.

*Hirsutella rhossiliensis* and *Hirsutella minnesotensis* (Ophiocordy-cipitaceae, Hypocreales, Ascomycota) are two representatives of nematode endoparasitic fungi (Liu et al. 2009; Sun et al. 2015). The complete mitogenome of *H. minnesotensis* has been reported (Zhang et al. 2015). Here, we present the complete mitogenome of *H. rhossiliensis* strain USA-87-5 (GenBank accession no. KU203675) isolated from parasitized second-stage juveniles of *Heterodera glycines* from a soybean field in Cottonwood county, Minnesota, America (44°2′24″ N, 94°55′48″ W). The specimen was deposited in the Herbarium of Microbiology, Academia Sinica (HMAS), while living culture was deposited at the China General Microbiological Culture Collection Center (CGMCC) (HMAS 246731; CGMCC 3.17882).

Whole-genome resequencing is performed on an Illumina HiSeq 2500-PE125 platform (Illumina Inc., San Diego, IL). A lane of 2 × 125 bp paired-end resequencing creates 3 915 863 000 clean reads based on our DNA sample. These reads are mapped with the Burrows-Wheeler Aligner (version 0.7.0) (Li & Durbin 2009) to the reference genome of *H. rhossiliensis* (Lai et al. unpublished) and assembled using SPAdes 3.1.1 (Bankevich et al. 2012) into 162 716 contigs. Then BLAST searches against known complete mitogenome of *H. minnesotensis* suggest 10 high similar fragments of total 49 257 bp with circularity. These fragments are annotated as two rRNA, 26 tRNA and 14 standard protein-coding genes using the MFannot tool (http://megasun.bch.umontreal.ca/cgi-bin/mfannot/MfannotInterface.pl). Gaps are filled by general PCR using a pair of specific primers designed by the software Primer3 (http://frodo.wi.mit.edu/primer3/) according to known flanking sequences.

The length of complete mitochondrial genome of *H. rhossiliensis* is determined to be 62 483 bp and contains 26 tRNAs, two rRNAs and 14 protein-coding sequences. The nucleotide composition of *H. rhossiliensi*s is 36.5% A, 12.8% C, 15.4% G and 35.3% T. The arrangement of 14 protein-coding genes and rRNAs is followed as *rnl*, *nad2*, *nad3*, *atp9*, *cox2*, *nad4L*, *nad5*, *cob*, *cox1*, *nad1*, *nad4*, *atp8*, *atp6*, *rns*, *cox3* and *nad6* identical to that of other common fungal mitochondrial genomes. Structural genes including 14 protein-coding genes, two rRNA and 26 tRNA genes cover 61.1% (38 180 bp) of the mitochondrial genome. The intergenic sequences have a total length of 24 303 bp occupying 38.9% of the genome. Intron sequences including introns of protein-coding genes account for 19.8% (12 395 bp) of the mitogenome.

The set of 26 tRNA genes codes for all 20 standard amino acids. Seventeen tRNA genes are adjacent to *rnl*, four tRNA genes approach to *rns* and five tRNA genes locate around three protein-coding genes (*cob*, *cox1* and *cox3*). Twelve introns invade six genes including *rnl* (one), *cob* (three), *cox1* (three), *cox3* (two), *nad1* (one) and *nad5* (two). These introns mainly belong to group I introns, but one (i.e. *nad5*-i1) is unclassified. Intronic proteins include ribosomal protein S3 and GIY-YIG/LAGLIDADG-type endonucleases or hypothetical proteins.

Phylogenetic analysis based on whole mitogenome sequences confirms *H. rhossiliensis* as a member of the fungal order Hypocreales. *Hirsutella rhossiliensis* is clustered together with *H. minnesotensis* and *Ophiocordyceps sinensis* within the family Ophiocordycipitaceae according to our phylogenetic analysis (Figure 1), with consistent taxonomic status according to phylogenetic analysis of nuclear genes of Hypocreales (Sung et al. 2007).

**Figure 1.**
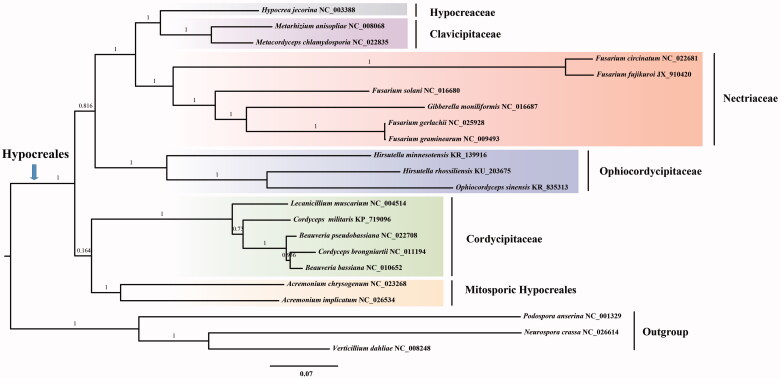
Phylogenetic analysis based on neighbour-joining method implemented in FastTree (Price et al. 2009) among 19 taxa of Hypocreales using whole mitogenome sequences. They are currently available in the GenBank database. The support values were shown above the nodes. *Podospora anserine*, *Neurospora crassa*, and *Verticillium dahliae* were used as the outgroups. Note that the accession number of *Ophiocordyceps sinensis* has not yet been released. The complete mitochondrial genome of *O. sinensis* has been reported (Li et al. 2015).
